# A refined panel of 42 microsatellite loci to universally genotype catarrhine primates

**DOI:** 10.1002/ece3.7069

**Published:** 2020-12-13

**Authors:** Franziska Trede, Niels Kil, James Stranks, Andrew Jesse Connell, Julia Fischer, Julia Ostner, Oliver Schülke, Dietmar Zinner, Christian Roos

**Affiliations:** ^1^ Cognitive Ethology Laboratory German Primate Center Leibniz Institute for Primate Research Göttingen Germany; ^2^ Primate Genetics Laboratory German Primate Center Leibniz Institute for Primate Research Göttingen Germany; ^3^ Department of Behavioral Ecology University of Göttingen Göttingen Germany; ^4^ Leibniz ScienceCampus Primate Cognition Göttingen Germany; ^5^ Research Group Primate Social Evolution German Primate Center Leibniz Institute for Primate Research Göttingen Germany; ^6^ Department of Microbiology Perelman School of Medicine University of Pennsylvania Philadelphia PA USA; ^7^ Department of Primate Cognition Georg‐August‐University Göttingen Germany; ^8^ Gene Bank of Primates German Primate Center Leibniz Institute for Primate Research Göttingen Germany

**Keywords:** apes, genotyping‐by‐sequencing, high‐throughput sequencing, Old World monkeys, simple tandem repeats

## Abstract

1. Microsatellite genotyping is an important genetic method for a number of research questions in biology. Given that the traditional fragment length analysis using polyacrylamide gel or capillary electrophoresis has several drawbacks, microsatellite genotyping‐by‐sequencing (GBS) has arisen as a promising alternative. Although GBS mitigates many of the problems of fragment length analysis, issues with allelic dropout and null alleles often remain due to mismatches in primer binding sites and unnecessarily long PCR products. This is also true for GBS in catarrhine primates where cross‐species amplification of loci (often human derived) is common.
2. We therefore redesigned primers for 45 microsatellite loci based on 17 available catarrhine reference genomes. Next, we tested them in singleplex and different multiplex settings in a panel of species representing all major lineages of Catarrhini and further validated them in wild Guinea baboons (*Papio papio*) using fecal samples.
3. The final panel of 42 microsatellite loci can efficiently be amplified with primers distributed into three amplification pools.
4. With our microsatellite panel, we provide a tool to universally genotype catarrhine primates via GBS from different sample sources in a cost‐ and time‐efficient way, with higher resolution, and comparability among laboratories and species.

## Graphical abstract

Even with genotyping‐by‐sequencing (GBS) of microsatellites issues with allelic dropout, null alleles and off‐target amplifications often remain due to poor primer design or using primers for cross‐species amplification. We re‐designed and validated a panel of 42 microsatellites providing a tool to universally genotype catarrhine primates via GBS from different sample sources in a cost‐ and time‐efficient way, with higher resolution, and comparability among laboratories and species.

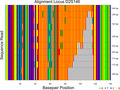

## 1 INTRODUCTION

Microsatellites have been and are still widely applied in various biological sciences including population genetics, kinship/pedigree analysis, human and wildlife forensics, linkage analysis, or disease association studies (e.g., Cunningham et al., 2001; Goodwin et al., 2011; Gulcher, 2012; Wasser et al., 2004). Population genetic information obtained by microsatellite genotyping is also important for monitoring wild populations in conservation contexts, for reintroduction programs or to refine captive breeding management (Arandjelovic & Vigilant, 2018; Norman et al., 2019). Microsatellites are also often the markers of choice to genetically characterize (wild) populations in order to determine degrees of population fragmentation and hybridization, dispersal patterns, mating systems, and reproductive success (e.g., Charpentier et al., 2012; de Moor et al., 2020; Ferreira da Silva et al., 2018; Kheng et al., 2017; McCarthy et al., 2020). The ongoing popularity of microsatellites is largely based on their high abundancy in animal genomes (Hamada et al., 1982; Tautz & Renz, 1984), the high levels of allelic diversity (Ellegren, 2000), and the possibility to amplify them across related species. Accordingly, microsatellites are preferred, for example over SNPs, because of their higher statistical power per locus and their cross‐species amplifiability, particularly when applied to small sample size datasets as typically found in forensic and kinship studies (Barbian et al., 2018; Guichoux et al., 2011).

However, traditional microsatellite genotyping via fragment length analysis (FLA) using polyacrylamide gel or capillary electrophoresis has several disadvantages, such as fragment size homoplasy, allele calling difficulties (stutter and split peaks, off‐target PCR products), laborious work and relatively high laboratory costs, as well as poor comparability of results among laboratories (De Barba et al., 2017; Guichoux et al., 2011; Pasqualotto et al., 2007). Even with attempts to improve PCR amplification and more accurate/reliable genotyping procedures (Arandjelovic et al., 2009; Buchan et al., 2005; Navidi et al., 1992; Sefc et al., 2003; Taberlet et al., 1996), many of the problems remained.

With microsatellite genotyping‐by‐sequencing (GBS) using high‐throughput sequencing technologies most of the difficulties can be mitigated (Barbian et al., 2018; Johannesen et al., 2017; Pimentel et al., 2018; Vartia et al., 2016). For instance, with GBS the exact length of the microsatellite alleles can be determined, which is a typical problem of FLA genotyping, particularly when alleles differ by only one basepair (bp) (Barbian et al., 2018; Vartia et al., 2016). Moreover, the nucleotide sequence is revealed so that cryptic alleles (alleles with the same length but containing a nucleotide variant) can be detected, resulting in an increased number of alleles and consequently greater statistical power per locus.

Nevertheless, problems with null alleles due to relatively large PCR products and allelic dropout as a result of primers binding in unconserved regions remain with GBS (Pompanon et al., 2005). As many microsatellites can be cross‐amplified in phylogenetically related species, primers designed for one species are often tested in related species and then applied if successfully amplified and informative (i.e., polymorph) (Barbara et al., 2007; De Barba et al., 2017). For example, various microsatellite loci characterized for humans can be successfully amplified in nonhuman catarrhine primates (Old World monkeys, apes) (Coote & Bruford, 1996; Ely et al., 1998; Kayser et al., 1996; Morin et al., 1998; Newman et al., 2002; Roeder et al., 2009; Smith et al., 2000) and have been used since then in numerous studies (e.g., Arandjelovic et al., 2014; Kopp et al., 2015; Minkner et al., 2018; Städele et al., 2019). Yet, attempts to reduce PCR product size or to adapt primers specifically to the study species have been rare (but see Bradley et al., 2000; Engelhardt et al., 2017; Inoue et al., 2016). Furthermore, various research groups use different panels of microsatellites preventing a direct comparison of results, particularly of measures such as genetic diversity and heterozygosity, which are important in a conservation context (Kolleck et al., 2013).

In our study, we aimed to establish a microsatellite panel to universally genotype catarrhine primates via GBS from different sample sources in a cost‐ and time‐efficient way, with higher resolution, and comparability among laboratories and species. Therefore, we screened a total of 269 microsatellite loci, widely targeted in catarrhine primates, and designed conserved primers for 45 loci based on available catarrhine genomes. We then tested the new microsatellite panel in ten primate species representing all major lineages of Catarrhini and further validated their applicability to low‐quality DNA samples using fecal samples of wild Guinea baboons (*Papio papio*).

## 2 MATERIAL AND METHODS

### 2.1 In silico selection of microsatellite loci

We screened 269 human microsatellite loci widely used in catarrhine population genetic studies. We extracted the human (GRCh38/hg38) sequence of each locus with 500 bp flanking regions from GenBank (https://www.ncbi.nlm.nih.gov/genbank/) and performed BLAT searches against the 16 available (status: 5 December 2018) nonhuman Catarrhini reference genomes (Table S1) using the UCSC (http://genome.ucsc.edu) or Ensembl (www.ensembl.org) genome browsers with standard settings. In addition, we checked the human sequence for repetitive elements (SINEs, LINES, etc.) in flanking regions using the RepeatMasker Web Server (http://www.repeatmasker.org/) with standard settings. We generated alignments for each locus containing the 16 nonhuman catarrhine species, the human, and the human repeat‐masked sequences with Muscle 3.8.31 (Edgar, 2004) in SeaView 4 (Gouy et al., 2010) and added published primer sequences to the alignments.

Loci were selected for further analysis if they fulfilled the following criteria: (a) primer binding sites are not in repetitive elements thus increasing locus‐specific amplifiability and reducing the risk of off‐target PCR products particularly in multiplex PCR reactions; (b) primer binding sites are conserved among catarrhines so that loci can be universally amplified in this taxonomic group with >180 species (Mittermeier et al., 2013); (c) the microsatellite motif is relatively short (max. 150 bp) to allow small amplicon size (max. 250 bp) and increase locus amplification success from degraded DNA samples, such as fecal samples; and (d) loci are evenly (1–3 loci per chromosome) distributed throughout the genome (using the genomes of *Homo sapiens*, *Nomascus leucogenys*, *Macaca mulatta*, and *Chlorocebus sabaeus* as reference) to avoid potential linkage problems.

For loci which passed the selection criteria, we designed new primers using Primer‐Blast (http://www.ncbi.nlm.nih.gov/tools/primer‐blast/). To allow for multiplexing, primers were designed to have similar annealing temperatures. Locus specificity of primers was checked by BLAT search against the 17 available catarrhine genomes. As primer binding sites were not always fully conserved among the 17 catarrhines, primers of 21 loci were designed with wobble positions. To simplify library preparation for GBS, we added adapter nucleotide sequences to the 5′ end of the locus‐specific primers (5′‐ACACTCTTTCCCTACACGACGCTCTTCCGATCT‐3′ to forward primers, 5′‐GTGACTGGAGTTCAGACGTGTGCTCTTCCGATCT‐3′ to reverse primers; locus‐specific primers are provided in Table S2).

### 2.2 Laboratory work

First, we tested in singleplex reactions for the locus specificity of selected primers and their universal applicability to catarrhine species in a panel representing all major lineages of catarrhines (Table 1). High‐quality DNA from a male of each of the ten species was obtained from the Gene Bank of Primates at the German Primate Center. PCRs were performed in total volumes of 25 µl containing 1× Qiagen Multiplex PCR Master Mix (Qiagen), 0.4 µM of each primer, and 50 ng genomic DNA. Cycling conditions comprised of 15 min at 95°C, 30 cycles each with denaturation at 94°C for 30 s, annealing at 57°C for 90 s and extension at 72°C for 90 s, and a final extension step of 10 min at 72°C. All reactions were run together with no‐template controls (NTCs) to check for contamination. PCR performance was checked on 2% agarose gels stained with ethidium bromide (Carl Roth GmbH). Sequencing of singleplex PCR reactions was omitted.

**TABLE 1:** Catarrhine species used to test the new microsatellite panel

Family	Subfamily	Tribe	Species
Hominidae	Homininae		*Pan troglodytes*
			*Gorilla gorilla*
	Ponginae		*Pongo abelii*
Hylobatidae			*Hylobates lar*
Cercopithecidae	Colobinae	Presbytini	*Trachypithecus obscurus*
			*Pygathrix nemaeus*
		Colobini	*Colobus guereza*
	Cercopithecinae	Cercopithecini	*Cercopithecus diana*
		Papionini	*Papio papio*
			*Macaca mulatta*

Next, we tested for the possibility of running multiplex PCR reactions to reduce overall laboratory work and costs. Therefore, we pooled either all 45 primer pairs in a single PCR reaction (1‐pool approach) or divided them into five PCR reactions each containing nine primer pairs (5‐pool approach) or three PCR reactions containing 18 and 2 × 12 primer pairs (3‐pool approach; for rationale of pooling and locus exclusion see Results section). Amplifications were conducted as described for the singleplex PCRs (same PCR set‐up, DNA samples, cycling conditions, NTCs), but with different primer concentrations (see Tables S3–S5 for pooling schemes and concentrations of single primers within pools). To minimize PCR errors, we ran PCR reactions in two independent replicates. PCR performance was again checked on 2% agarose gels. Replicate PCR products (including the NTCs) were pooled and then cleaned with the MinElute PCR Purification Kit (QIAGEN). DNA concentrations were measured with a Qubit 3.0 (Thermo Fisher Scientific) and 100 ng were subjected to indexing PCR. Indexing PCR was performed in total volumes of 25 µl containing 1× KAPA HiFi HotStart ReadyMix (Roche), 0.4 µM of each indexing primer and 100 ng purified PCR product. Cycling conditions comprised of 45 s at 98°C, 4 cycles each with denaturation at 98°C for 15 s, annealing at 62°C for 30 s and extension at 72°C for 30 s, and a final extension step of 1 min at 72°C. Subsequently, indexed PCR products were purified with the MinElute PCR Purification Kit (Qiagen) and ran on a Bioanalyzer 2100 (Agilent) to check for PCR performance and molarity. Libraries were diluted to a final concentration of 10 nM and then pooled and sequenced with 51 cycles forward and 251 cycles reverse on Illumina's MiSeq desktop sequencer.

To check for Mendelian inheritance and whether our new microsatellite panel is also applicable to low‐quality and low‐quantity DNA as typically extracted from fecal samples (Monteiro et al., 1997; Perry et al., 2010), we tested our panel in 12 fecal samples of wild Guinea baboons. The samples comprised of six males and two “families” each composed of a male, a female, and their known offspring. DNA from these 12 specimens was previously genotyped via FLA at 24 microsatellite loci (Dal Pesco, 2019). The amplification procedure and follow‐up steps for the applied 3‐pool approach were the same as described above, but the number of cycles in the initial amplification was increased to 40, the total DNA amount was increased to 200 ng, and each PCR was performed in triplicates (Barbian et al., 2018).

### 2.3 Bioinformatic analysis

The data analysis was performed using the software package CHIIMP v.3.0.0 (Barbian et al., 2018). The raw data (FASTQ files) as well as all input files (config‐file, sample‐file, locus‐attributes‐file) are available in the online supplement resources. As our microsatellite panel included several di‐repeat loci, which stutter more frequently than tetra‐repeats, we increased the stutter count ratio to 0.70 (stutter.count.ratio_max: 0.7). We further implemented a broad range of possible allele lengths in the locus attributes by setting the length buffer to 100 bp. This ensured the inclusion of all tested species even if the allele sizes at a given locus varied between species according to the available reference genomes. The minimum number of reads per locus was set to 100 (counts.min: 100). All other parameters were set to default.

With the current version of CHIIMP, wobble positions in primer sequences cannot be accounted for. Hence, for loci with a wobble position in a primer sequence, alternative nucleotides of the wobble are erroneously recognized as different alleles. Moreover, the repeat motif needs to be specified in CHIIMP, but as repeat motifs can vary in the investigated species, correct (orthologous) reads remain unrecognized for some species if CHIIMP is fed with a wrong repeat motif. Due to these reasons, the output for all loci was checked manually and corrected if needed. Additionally, we screened the processed reads for the general level of amplification per locus and the occurrence of PCR artifacts (off‐target amplification, primer dimer, false primer pairings, etc.).

## 3 RESULTS

### 3.1 In silico selection of microsatellite loci

In total, 217 of the 269 investigated loci were not optimal for microsatellite genotyping of catarrhines. For 147 of them, one or both primer binding sites or the complete locus were located in repetitive elements. This increases the likelihood to amplify various off‐target PCR products, particularly in multiplex settings when many primers that can bind multiple times in the respective genome are combined in a single PCR reaction. For an additional 32 loci, we could not find conserved primer binding sites near the microsatellite and a further 15 loci contained relatively long microsatellite repeat regions for one or more species, resulting in long PCR products (>250 bp). Longer PCR products are often difficult to generate if only degraded DNA material is available and can result in null alleles. Further problems included, for instance, the location of loci directly next to each other on the same chromosome and thus increasing the risk of linkage. Additionally, double entries of loci under different names or gaps in some of the reference genomes (especially for Y‐chromosomal loci) impeded the screening process. A full list of screened loci including the respective reasons for their exclusion is provided in Table S6. Of the 52 loci which fulfilled our criteria, we selected 45 (1–3 loci per human chromosome including gonosomes) for downstream analyses. The chromosomal locations of the chosen loci in the genomes of *H. sapiens*, *N. leucogenys*, *M. mulatta,* and *C. sabaeus* are provided in the supplement (Table S2). We found no indication for the presence of linkage between any of the loci in any of the four investigated species (minimal distance between two loci 5.35 million bp).

The newly designed primers for the 45 loci (consisting of di‐, tri‐, and tetra‐repeats) amplify PCR products between 56–215 bp (according to available genome data; Table S2). Compared to the original published primers, we were able to reduce PCR product sizes by 2–225 bp (mean 75.9 bp) in 37 loci whereas for five loci, the new primers amplify a moderately longer fragment (elongation by 2 – 15 bp; mean 7.6 bp). PCR product size for the remaining three loci did not change. As primer binding sites were not always perfectly conserved among the 17 investigated catarrhine reference genomes, primers for 21 loci contain wobble positions. Mismatches in primer binding sites, found only in a few (1–2) of the investigated species, were neglected in primer design and probably result in less efficient or no amplification of the respective locus in the given species (0–12 loci with mismatches per species, mean 3.4; Table S2).

### 3.2 Singleplex PCR test

Singleplex PCR reactions of the 45 loci in ten species representing all major lineages of catarrhines were run on agarose gels and resulted, for all loci and species, in PCR products within the expected size range with no signs of amplifying any off‐target PCR products (data not shown). Thus, locus specificity and universal applicability of our primer set to catarrhine primates was indicated.

### 3.3 Multiplexing approaches

Sequenced alleles ranged in size from 71 bp (D3s1768) to 211 bp (D12s372) and nine loci contained cryptic alleles in at least one species (Tables S7–S10). The level of amplification and obtained sequence reads varied across samples/species and loci in all three approaches. The amplification of all loci in one pool (1‐pool approach) was least effective, resulting in the lowest number of amplified loci (mean 25.9) and alleles (mean 36.9; Table 2). In some cases, the reason for allelic dropouts could be attributed to wrong primer pairing/primer mismatches (primer dimer or off‐target amplification of short products). Most loci amplified less efficiently than in the other two approaches and some (*N* = 11) failed to amplify at all. Only nine loci recovered the same number of alleles as in the 5‐pool approach. Interestingly, even though the number of amplified alleles was reduced from a mean of 60.1 to 36.9 compared to the 5‐pool approach, the level of heterozygosity was not affected to the same extent with a reduction from 49.8% to 43.5% (Table 2).

**TABLE 2:** Number of amplified loci and alleles, as well as the level of heterozygosity per species generated in three approaches with high quality DNA (blood) and degraded DNA from fecal samples

	High‐quality DNA			Degraded DNA
	5‐pool approach (45 loci)	1‐pool approach (45 loci)	3‐pool approach (42 loci)	3‐pool approach (42 loci)
Mean number of loci amplified per sample/species (range)	40.2 (37–43)	25.9 (21–32)	37.8 (33–41)	38.8 (35–41)
Mean number of alleles amplified per sample/species (range)	60.1 (53–69)	36.9 (31–45)	55.5 (46–68)	52.2 (47–56)
Mean level of heterozygosity per sample/species (range)	49.8% (23.3%–69.4%)	43.5% (22.6%–71.4%)	47.1% (19.5%–66.7%)	34.3% (23.1%–47.4%)^a^ 46.3% (31.0%–64.3%)^b^

^a^ Including all 42 loci.

^b^ Including only the 32 loci that were polymorphic in the study species.

The best results, that is, the highest amplification levels for loci (mean 40.2) and alleles (mean 60.1), were generated applying the 5‐pool approach (Table 2). Nevertheless, we observed again primer dimers and short off‐target PCR products potentially as a result of interacting primers from different loci. Moreover, three loci (D11s1366, D12s67.2, and D15s1007) neither amplified in the 1‐pool nor in the 5‐pool approach and were excluded from further testing. To further improve amplification success and to reduce primer interactions among primers of different loci (based on the knowledge obtained from the 1‐pool and 5‐pool approaches), we distributed the 42 remaining loci into three amplification pools containing 18, 12 and 12 loci, respectively (Table S5). Using the 3‐pool approach, we were able to largely minimize primer interactions, but amplification success for loci (mean 37.8) and alleles (mean 55.5) per species was slightly reduced compared to the 5‐pool approach, but higher than in the 1‐pool approach (Table 2). The reduced amplification success was due to allelic dropouts of single alleles or whole loci in some species (see Table S9).

### 3.4 Degraded DNA samples

For the degraded DNA samples, we applied the 3‐pool approach as this represented the best compromise between amplification efficiency and laboratory effort and costs (see Results Multiplex approaches). The amplification from fecal samples was successful except for four (out of 42) loci (two autosomal and two gonosomal loci; Table S10). The number of loci and alleles amplified per sample was comparable to the results obtained from high‐quality DNA samples (Table 2). However, 10 of the 42 amplified loci were monomorphic in our *P. papio* population, that is, all twelve individuals showed the same allele. The remaining 32 loci showed a level of 46.3% heterozygosity (Table 2).

All autosomal loci were in accordance with Mendelian inheritance besides D7s503 and D13s1291. For D7s503, the two alleles with the highest read counts for male MRX (99/109) did not match one of the two alleles of his offspring THL (111/113; mother MMI: 103/113). A closer look at the data revealed that MRX also had many reads for allele 111 (only 23 reads less than for allele 109), indicating that the allele 109 of MRX was likely an overamplified stutter sequence. A genotype with the allele combination 99/111 also corresponds to the genotype derived from FLA for this locus (152/164; 12bp distance between alleles, based on >20 amplifications; Dal Pesco, 2019). For D13s1291, the two alleles of male MLK (130b/132b) did not match with his offspring PTC (130a/132a; mother LCY: 128/130a). MLK was the only individual with these two potential cryptic alleles (each with a G→A point mutation) and further showed reads with 130 and 132 bp length without this mutation. At this point, we can neither exclude the occurrence of a PCR artifact in this particular case, nor that this locus does not follow the rules of Mendelian inheritance.

## 4 DISCUSSION

From a set of 269 microsatellite loci widely applied in catarrhine primates, we selected a total of 45 loci that can be universally applied to genotype catarrhine primates. Due to the relatively small amplicon sizes, even low‐quality DNA could be genotyped and since the selected loci were evenly distributed throughout the genome (at least according to the human genome), the risk of linkage was significantly reduced. Moreover, our panel could be multiplexed to a great extent. The testing of different multiplex settings revealed that a 5‐pool approach produced the best result, but that a 3‐pool approach containing one pool of 18 and two of 12 loci is the best compromise between locus amplification efficiency and laboratory effort and costs.

We tested the panel with high‐quality DNA samples from all major lineages of catarrhines in multiplex settings and revealed successful amplification rates of 33 to 41 (average 38) loci per species (Table 2). We additionally showed the applicability of the 3‐pool approach to degraded DNA samples such as fecal samples, which is a common material in many noninvasive wildlife studies (Carroll et al., 2018; Waits & Paetkau, 2005). The results for fecal samples were similar to the results of the high‐quality samples with respect to the mean number of loci and alleles amplified per sample (Table 2). All loci, besides D13s1291, were in accordance with Mendelian inheritance, demonstrating the suitability of the new microsatellite panel for kinship and relatedness analyses. To ensure high‐quality genotypes from fecal samples, and depending on DNA quality, further adaptions to the protocol might be necessary. Quantifying the endogenous DNA content via quantitative PCR prior to genotyping will help to select only those samples with sufficient endogenous DNA content (e.g., >25 pg endogenous DNA as suggested by Barbian et al., 2018). Additionally, multiple samples per individual can be analyzed or the number of PCR replicates per sample can be increased.

Through multiplexed GBS, cryptic alleles can be detected (Barbian et al., 2018; Sarhanova et al., 2018; Vartia et al., 2016), and even in our test panel of only ten catarrhine species with one individual each, we found cryptic alleles at nine loci (Tables S7–S10). Although our results are based on only two or three replicates per approach (depending on the sample type) and hence should be interpreted with caution, we are confident that these alleles are indeed cryptic alleles and not PCR artifacts. In case of PCR artifacts, we would expect mixed sequence reads showing more than two alleles or highly imbalanced sequence read counts for the “true allele” and the “artifact allele,” as it is highly unlikely that the same PCR artifact occurs in all replicates. As more individuals per species get tested, the number of cryptic alleles will most likely increase and provide further accuracy and a higher statistical power of our panel.

Another advantage of GBS is that the resulting genetic data, in form of allele sequences, are independent of the used sequencing platform. Thus, data produced by different laboratories can be easily shared and compared. By applying validated bioinformatics pipelines, such as the CHIIMP pipeline (Barbian et al., 2018), one can further ensure that the resulting data are reproducible and less prone to arbitrary allele calling by different researchers while still allowing the customization of, for example, filtering parameters to fit different datasets.

Although we recommend the 3‐pool approach, the amplification success of individual loci can be improved, for example, by amplifying all loci in individual reactions and then pooling before or after the indexing PCR. However, this would largely increase workload in the laboratory and costs. It is also important to check which loci are polymorphic in the species of interest, so that monomorphic loci can be excluded from large‐scale population genetic investigations. Likewise, as several species exhibit mismatches in primer binding sites (0–12 loci with mismatches per species), primer design for a given species can be adjusted and optimized, which becomes easier to do with an increasing number of sequenced catarrhine genomes.

In summary, with our microsatellite panel, we provide a tool to universally genotype catarrhine primates via GBS from samples of varying DNA quality in a cost‐ and time‐efficient way, with higher resolution, better comparability among laboratories, and largely mitigated problems of traditional FLA.

## CONFLICT OF INTEREST

The authors declare no conflict of interest.

## AUTHOR CONTRIBUTION

**Franziska Trede:** Investigation (equal); Writing‐original draft (equal); Writing‐review & editing (equal). **Niels Kil:** Investigation (equal); Writing‐review & editing (equal). **James Stranks:** Investigation (equal); Writing‐review & editing (equal). **Andrew Jesse Connell:** Software (lead); Writing‐review & editing (equal). **Julia Fischer:** Funding acquisition (equal); Writing‐review & editing (equal). **Julia Ostner:** Funding acquisition (equal); Writing‐review & editing (equal). **Oliver Schülke:** Funding acquisition (equal); Writing‐review & editing (equal). **Dietmar Zinner:** Writing‐original draft (equal); Writing‐review & editing (equal). **Christian Roos:** Conceptualization (equal); Investigation (supporting); Writing‐original draft (equal); Writing‐review & editing (equal).

## Supporting information

Appendix S1

## Data Availability

The sequencing data have been submitted to the NCBI Sequence Read Archive (SRA) under BioProject number PRJNA672243 (http://www.ncbi.nlm.nih.gov/bioproject/672243). Input files for the bioinformatics analysis (config‐, sample‐, and locus‐attributes‐files) are available online in the supporting information (Appendix S1).
